# Small renal masses in Latin-American population: characteristics and prognostic factors for survival, recurrence and metastasis – a multi-institutional study from LARCG database

**DOI:** 10.1186/s12894-020-00649-8

**Published:** 2020-07-02

**Authors:** Thiago Camelo Mourão, Diego Abreu, Gustavo F. Carvalhal, Guillermo Gueglio, Walter H. da Costa, Vinicius Fernando Calsavara, Luis Meza-Montoya, Rubén G. Bengió, Carlos Scorticati, Ricardo Castillejos-Molina, Francisco Rodríguez-Covarrubias, Ana María Autran-Gómez, José Gadu Campos-Salcedo, Alejandro Nolazco, Carlos Ameri, Hamilton Zampolli, Raúl Langenhin, Diego Muguruza, Marcos Tobias Machado, Pablo Mingote, Jorge Clavijo, Lucas Nogueira, Omar Clark, Agustín R. Rovegno, Fernando P. Secin, Ricardo Decia, Gustavo C. Guimarães, Sidney Glina, Oscar Rodríguez-Faba, Joan Palou, Stenio C. Zequi

**Affiliations:** 1 A.C. Camargo Cancer Center, Rua Antônio Prudente 211, Liberdade, São Paulo, 01509-010 Brazil; 2 Pasteur Hospital, Montevideo, Uruguay; 3 São Lucas Hospital – PUCRS, Porto Alegre, Brazil; 4 Italian Hospital, Buenos Aires, Argentina; 5 Epidemiology and Statistics Department, International Research Center, A.C.Camargo Cancer Center, São Paulo, Brazil; 6 Instituto Nacional de Enfermedades Neoplásicas, Lima, Peru; 7 Urological Center Profesor Bengió, Cordoba, Argentina; 8 Clinics Hospital José de San Martín, Buenos Aires, Argentina; 9 Instituto Nacional de Ciencias Médicas y Nutrición Salvador Zubirán (INCNSZ), Mexico City, Mexico; 10 Jiménez Díaz Foundation, Madrid, Spain; 11 Military Hospital, Mexico City, Mexico; 12 British Hospital, Buenos Aires, Argentina; 13 German Hospital, Buenos Aires, Argentina; 14 Instituto Arnaldo Vieira de Carvalho, São Paulo, Brazil; 15 Corporación Médica de Paysandú (COMEPA), Paysandu, Uruguay; 16 ABC Medical School, São Paulo, Brazil; 17 Policlinico Neuquén, Neuquen, Argentina; 18 Clinics Hospital, Montevideo, Uruguay; 19 Federal University of Minas Gerais, Belo Horizonte, Brazil; 20 Military Hospital, Montevideo, Uruguay; 21 Centro de Educación Médica e Investigaciones Clínicas (CEMIC), Buenos Aires, Argentina; 22 San Lázaro Foundation and FUNDES, Buenos Aires, Argentina; 23 Surgical Oncology Coordinator at Beneficencia Portuguesa Hospital, São Paulo, Brazil; 24 Ipiranga Hospital, São Paulo, Brazil; 25 Puigvert Foundation, Barcelona, Spain; 26 National Institute for Science and Technology in Oncogenomics and Therapeutic Innovation, AC Camargo Cancer Center, São Paulo, Brazil

**Keywords:** Kidney cancer, Nephron-sparing surgery, Latin America, Renal cell carcinoma, Nephrectomy

## Abstract

**Background:**

To evaluate demographic, clinical and pathological characteristics of small renal masses (SRM) (≤ 4 cm) in a Latin-American population provided by LARCG (Latin-American Renal Cancer Group) and analyze predictors of survival, recurrence and metastasis.

**Methods:**

A multi-institutional retrospective cohort study of 1523 patients submitted to surgical treatment for non-metastatic SRM from 1979 to 2016. Comparisons between radical (RN) or partial nephrectomy (PN) and young or elderly patients were performed. Kaplan-Meier curves and log-rank tests estimated 10-year overall survival. Predictors of local recurrence or metastasis were analyzed by a multivariable logistic regression model.

**Results:**

PN and RN were performed in 897 (66%) and 461 (34%) patients. A proportional increase of PN cases from 48.5% (1979–2009) to 75% (after 2009) was evidenced. Stratifying by age, elderly patients (≥ 65 years) had better 10-year OS rates when submitted to PN (83.5%), than RN (54.5%), *p* = 0.044. This disparity was not evidenced in younger patients. On multivariable model, bilaterality, extracapsular extension and ASA (American Society of Anesthesiologists) classification ≥3 were predictors of local recurrence. We did not identify significant predictors for distant metastasis in our series.

**Conclusions:**

PN is performed in Latin-America in a similar proportion to developed areas and it has been increasing in the last years. Even in elderly individuals, if good functional status, sufficiently fit to surgery, and favorable tumor characteristics, they should be encouraged to perform PN. Intending to an earlier diagnosis of recurrence or distant metastasis, SRM cases with unfavorable characteristics should have a more rigorous follow-up routine.

## Background

Comprehensive approach of renal cell carcinoma (RCC) is a priority in the main cancer centers. Increasing trend in incidence, particularly of incidentally detected small renal masses (SRM), has not been clearly associated to a significant decrease in global mortality. According to the SEER (Surveillance, Epidemiology, and End Results) database, the estimated number of new cases of RCC in the United States in 2019 is 73,820. RCC represents about 4% of all new cancer cases and about 2.5% of all cancer deaths [1–3].

Latin America (LA) represents a large area with about 8.5% of the world population, most of them living in developing countries. GLOBOCAN estimated the RCC incidence and mortality in LA in 4.4/100,000 and 1.5/100,000, respectively [4]. Recently, an elegant study suggested that in 2030, LA countries (Brazil and Ecuador) will experiment the highest burden of increase of new cases in both genders [5]. RCC data in LA are scarce and, over time, miscegenation has impacted this population in a singular ethnicity, which could make unreliable extrapolating biomolecular profiles and guided therapies already stablished in developed world populations [6].

Nephron-sparing surgery (NSS) is the standard of care for SRM, providing equivalent oncological control and a decrease in the non-cancer related mortality [7, 8]. Besides that, even with the improvement of minimally-invasive and robotic techniques, large series continue to evidence a high proportion of radical nephrectomy (RN) in this setting [7, 9–11].

Considering the paucity of information about RCC in LA, the aims of this study were to review the LARCG (Latin-American Renal Cancer Group) database to evaluate demographic, clinical and pathologic features of SRM in this population, correlating the impact in prognosis and survival in comparison to literature.

## Methods

### Data source

This is a multi-institutional retrospective cohort study involving the registries from 28 institutions in LA and Spain, members of LARCG [6]. Data were collected from patient charts and pathological reports from each institution with a total of 6039 cases of RCC operated from 1979 to 2016 in Argentina, Bolivia, Brazil, Chile, Mexico, Peru, Uruguay, and two Spanish centers. Institutional Review Board of Fundação Antônio Prudente approved this protocol (number 2.478.489, CAAE: 71749917.3.0000.5432/January 30th^.^ 2018) and the study did not interfere with the treatment or follow-up of the patients.

### Study population and variables definition

We selected patients with renal tumors up to 4 cm on pathological reports and no evidence of distant metastasis at presentation. We found 1523 patients with these characteristics which were stratified by histologic subtype criteria in clear cell, papillary, chromophobe or others (collecting duct carcinoma, TTFE3 gene, unclassified and mixed histologies) and by surgical treatment: partial nephrectomy (PN) or RN. Firstly, we excluded patients with benign histology and those with missing values for histologic subtype. Afterwards, non-surgical management cases were excluded.

Demographic, clinical and pathological variables selected were age, gender, race, body mass index (BMI), smoking status, ECOG (Eastern Cooperative Oncology Group) Performance Status, Karnofsky and ASA (American Society of Anesthesiologists) classifications, symptoms at presentation, tumor size (≤ 3 cm or >  3 – 4 cm), positive surgical margins (PSM), nuclear Fuhrman grade (low or high grade), intratumoral necrosis, sarcomatoid component, extracapsular extension (ECE), multifocality or bilaterality, and admission period (before or after 2009). Patients were considered symptomatic at presentation if they had hematuria, local pain, palpable mass, paraneoplastic syndrome, neurological symptoms, acute onset varicocele and constitutional symptomatology. Nuclear grade was just established for clear cell and papillary RCC [12]. ECE was considered in cases presenting disease in perinephric fat, hilar fat and/or in renal pelvis.

Follow-up was established from the date of surgery to the date of death or to the latest follow-up. Treatment indications and follow-up routine were determined at the discretion of each center. Overall deaths considered deaths due to the renal cancer and deaths from other causes. Local recurrence was defined as recurrence in the renal fossa after RN, and demonstration of recurrent enhancement within the tumor bed or enhancing suspicious lesion in the ipsilateral kidney after PN.

Authors used a multiple imputation method only in variables with a limited proportion of missing data [13]. The data were assumed to be missing completely at random (MCAR) and a method of predictive mean machine (PMM) was adopted [14]. Variables with more than 50% of missing data and non-attributable characteristics (e.g., race, and histological subtype) were not submitted to the imputation method. This method was used for the following variables: perinephric fat invasion (24% of missing data), smoking status (32% of missing data), intratumoral necrosis (41% of missing data), weight (42% of missing data), and ECOG performance status (44% of missing data).

### Statistical analysis

Continuous variables were expressed as mean, standard deviation, median and interquartile range. Categorical variables were expressed as absolute relative frequencies. They were compared with a Pearson’s chi-squared test or a Fisher’s exact test, when appropriate. Student’s *t*-test was used to compare continuous variables. Kaplan-Meier curves and log-rank tests were used in 10-year overall survival (OS) estimation, according age, tumor size and surgery performed. Considering that some patients underwent the surgical treatment at a referral center and then return to their communities, survival analysis were performed only for patients with follow-up ≥ 12 months. Univariable and multivariable logistic regression analysis were used to predict independent factors for local recurrence or distant metastasis. Related to these outcomes, it was considered the entire period, including all the patients regardless of the available follow-up time. The analysis was conducted using IBM SPSS®software v.24 (IBM Corp. Armonk, NY, USA) and the packages R-Software v. 3.5 for graphical analysis. The significance level of the tests were fixed at 0.05.

## Results

A total of 1008 cases had a defined malignant histologic subtype, being 651 cases (64.6%) of clear cell RCC, 60 (6%) of papillary RCC (types I and II), 238 (23.6%) of chromophobe RCC, and 59 (6%) of other histologies. PN and RN were performed in 897 (66%) patients and 461 (34%), respectively. The proportion of PN cases increased from 48.5% (up to 2009) to almost 75% after that year. Table 1 summarizes the main clinical and pathological characteristics.

**Table 1:** Comparison of clinical and pathological data between patients with small renal masses who performed partial or radical nephrectomy

Variable Category	Partial Nephrectomy (***n*** = 897)	Radical Nephrectomy (***n*** = 461)	***p*** Value *
**Year of the surgery**			
Up to 2009 n (%)	225 (48.5)	239 (51.5)	**< 0.001**
Since 2010 n (%)	637 (74.9)	214 (25.1)	
**Age (years)**			
Mean (SD)	57.9 (12.7)	61.2 (12.8)	**< 0.001**
Median (Q1 - Q3)	59 (50–67)	63 (53–71)	
**BMI (Body Mass Index)**			
Mean (SD)	27.6 (4.8)	27.5 (4.9)	0.661
Median (Q1 – Q3)	26.9 (24.2–29.9)	26.7 (24.2–30)	
**Blood Transfusion** n (%)	107 (12.6)	43 (10.5)	0.317
**Serum Creatinine -** mg/dL			
Baseline - Mean (SD)	1.02 (0.61)	1.23 (1.23)	**0.011**
Median (Q1 – Q3)	0.96 (0.86–1.1)	1.0 (0.8–1.2)	
Post-operative - Mean (SD)	1.13 (0.51)	1.50 (1.50)	**0.003**
Median (Q1 – Q3)	1.1 (0.9–1.3)	1.3 (1.1–1.5)	
**Operation time –** min			
Mean (SD)	174.4 (76.6)	197.3 (86.8)	**< 0.001**
Median (Q1 – Q3)	120 (90–180)	180 (120–240)	
**ECOG**			
0–1 n (%)	862 (96.1)	437 (94.8)	0.329
≥ 2 n (%)	35 (3.9)	24 (5.2)	
**Karnofsky**			
≤ 80 n (%)	74 (8.2)	46 (10)	0.336
> 80 n (%)	823 (91.8)	415 (90.0)	
**ASA classification**			
1–2 n (%)	767 (85.5)	359 (77.9)	**0.001**
≥ 3 n (%)	130 (14.5)	102 (22.1)	
**Renal tumor**			
Exophytic tumor	236 (62.8)	140 (37.2)	**< 0.001**
Totally endophytic tumor	19 (26.0)	54 (74.0)	
≤ 3 cm	538 (74.8)	181 (25.2)	**< 0.001**
3.1 - 4 cm	261 (53.0)	231 (47.0)	
**Extracapsular extension** n (%)			
Yes	51 (6.9)	87 (20.8)	**< 0.001**
No	686 (93.1)	332 (79.2)	
**Surgical Margins** n (%)			
Positive	42 (4.7)	7 (1.6)	**0.008**
Negative	844 (95.3)	422 (98.4)	
**Nuclear grade**			
Low grade n (%)	685 (76.4)	330 (71.6)	0.064
High grade n (%)	212 (23.6)	131 (28.4)	
**Sarcomatoid component** n (%)			
Yes	2 (0.6)	3 (1.2)	0.654
No	354 (99.4)	253 (98.8)	
**Tumoral necrosis** n (%)			
Yes	104 (11.6)	77 (16.7)	**0.011**
No	793 (88.4)	384 (83.3)	
**Multifocality** n (%)			
Yes	41 (5.0)	33 (7.7)	0.070
No	782 (95.0)	395 (92.3)	
**Locoregional recurrence** n (%)			
Yes	19 (4.4%)	26 (7.2%)	0.126
No	410 (95.6%)	334 (92.8%)	

* *p*-Value < 0.05 considered statistically significant. Pearson’s chi-squared test or Fisher’s exact test were used in categorical variables when appropriate. Student’s t-test was used in continuous variables

Regarding surgical aspects of PN cases, open and minimally-invasive approaches (laparoscopic, retroperitoneoscopic or robot-assisted) were performed in 47.9 and 50.4%, respectively. Median blood loss was 200 mL (5 – 2500 mL), median warm ischemia time was 20 min, and median hospital stay was 4 days (1–37).

The median follow-up time was 24 months (range: 0–289). We reported 72 (4.7%) overall deaths and 30 (2.0%) cancer-specific deaths. Local recurrence occurred in 49 (6.1%) from 804 cases and metastasis during follow-up, in 10 (1.7%) from 588 evaluable cases.

**Fig. 1:**
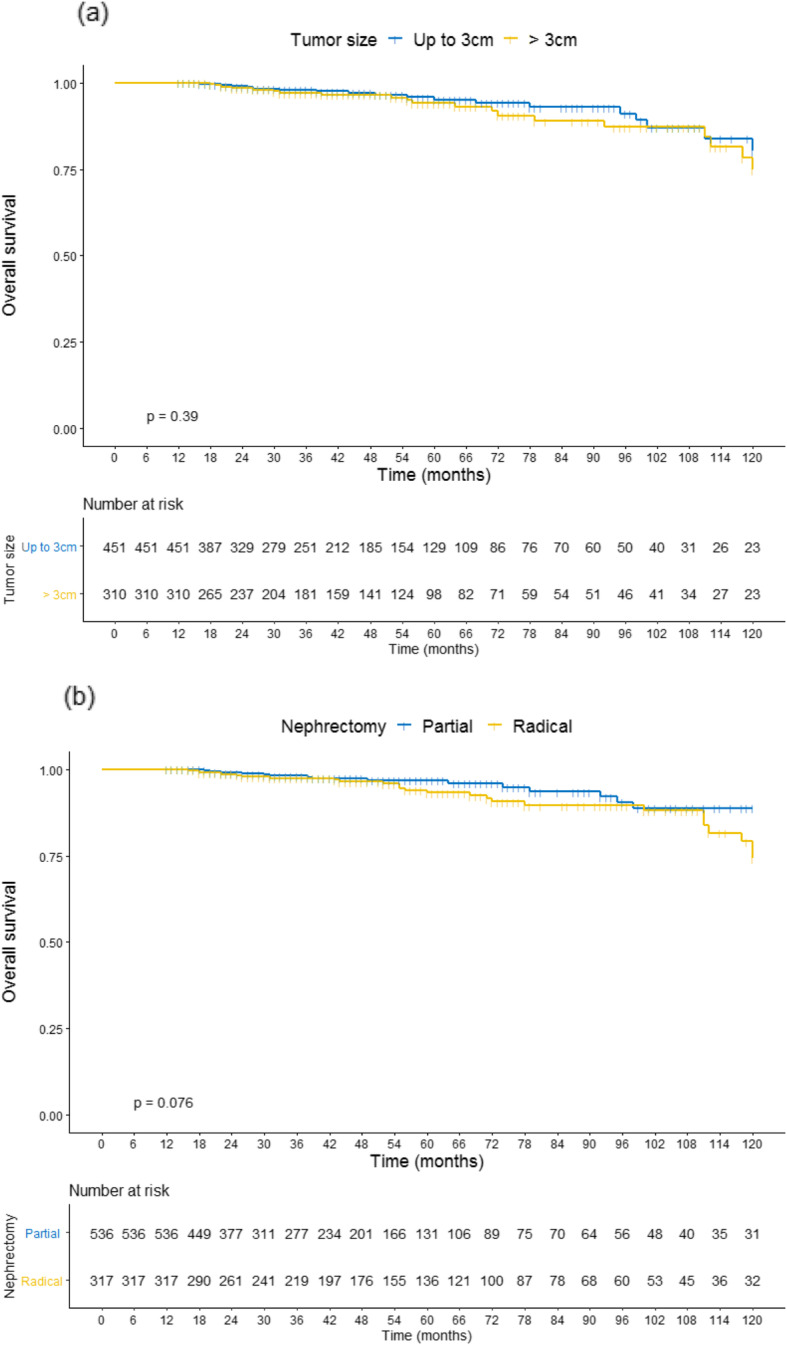
Kaplan-Meier curve for 10-year overall survival (OS) in evaluable patients according to the tumor size (**a**) and the surgical treatment (**b**)

There were no significant differences in 10-year OS rates comparing patients with SRM ≤ 3 cm or >  3 cm. Similarly, OS rates were not different between patients underwent PN or RN. The 5-year and 10-year OS rates for SRM ≤ 3 cm were 95.1 and 80.3%, respectively. For SRM > 3 cm, 5-year and 10-year OS rates were 94.1 and 74.8%, respectively (Fig. 1-a). Patients underwent PN presented the 5-year and 10-year OS of 96.9 and 88.8%, respectively; whereas RN cases achieved 93.2 and 74.2%, respectively (Fig. 1-b).

**Fig. 2:**
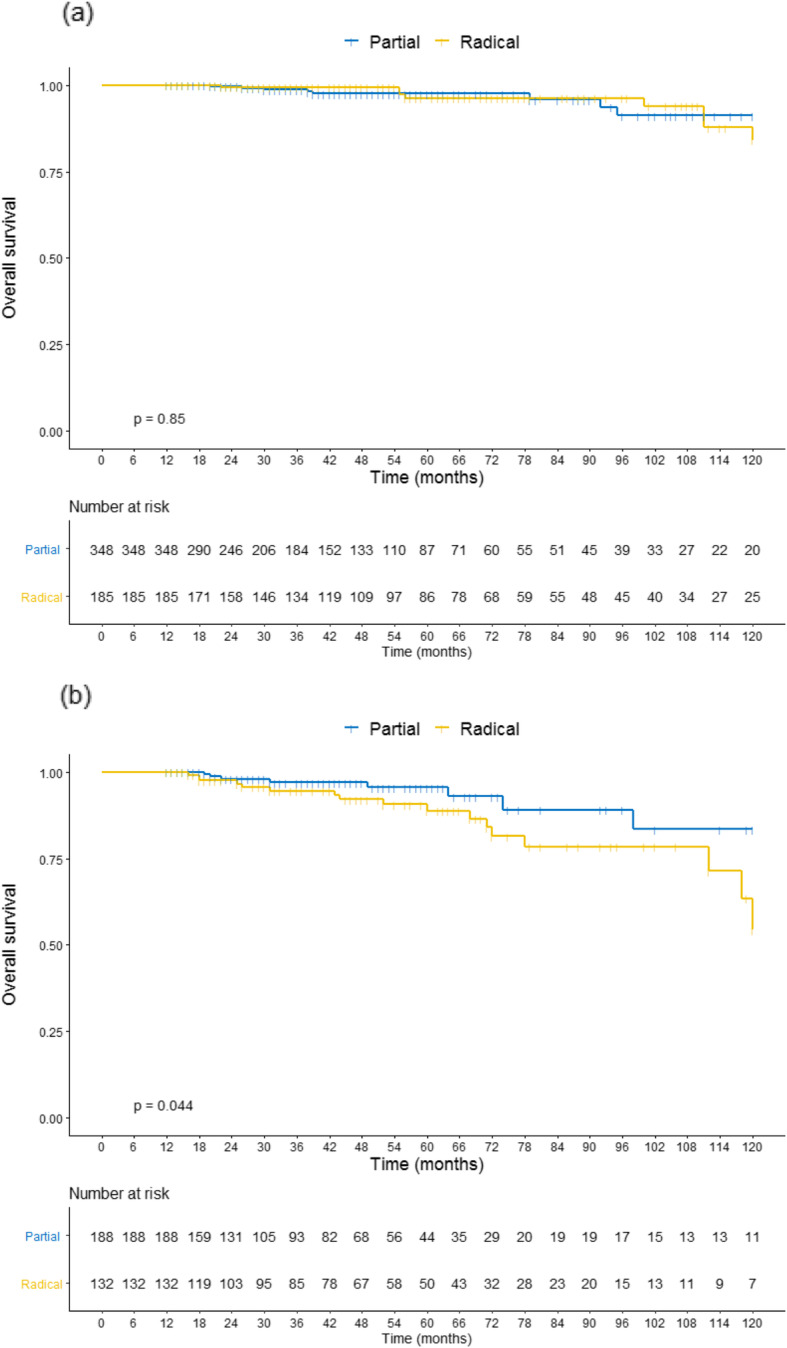
Kaplan-Meier curve for 10-year overall survival (OS) comparing patients submitted to PN or RN according to the group of age. **a** <  65 years **b** ≥ 65 years

Stratifying by age, elderly patients (≥ 65 years) had better 10-year OS rates when submitted to PN (83.5%), than RN (54.5%), *p* = 0.044 (Fig. 2-b). This disparity was not evidenced in younger patients (Fig. 2-a). Elderly group presented more comorbidities, ASA ≥ 3, larger SRM, and a higher proportion of RN than younger. A crosstab analysis considering younger and elderly patients is presented in Table 2.

**Table 2:** Comparison of clinical and surgical data between younger (< 65 years) and elderly patients (≥ 65 years) with small renal masses

	< 65 years (***n*** = 949)	≥ 65 years (***n*** = 574)	***p*** Value *
**Gender**			
Male n (%)	644 (68.1)	378 (66.3)	0.496
Female n (%)	301 (31.9)	192 (33.7)	
**Comorbidities** n (%)			
Yes	552 (61.3)	387 (72.6)	**< 0.001**
No	349 (38.7)	146 (27.4)	
Hypertension n (%)	115 (18.0)	128 (37.8)	**< 0.001**
**Obesity** n (%)			
Yes	229 (24.9)	131 (23.6)	0.634
No	691 (75.1)	423 (76.4)	
**Smoking** n (%)			
Yes	339 (35.7)	190 (33.1)	0.324
No	610 (64.3)	384 (66.9)	
**Symptomatic at presentation** n (%)			
Yes	442 (46.6)	260 (45.3)	0.666
No	507 (53.4)	314 (54.7)	
**Serum Creatinine - mg/dL**			
Baseline - Mean (SD)	1.04 (0.81)	1.17 (0.92)	**0.045**
Median (Q1 – Q3)	0.96 (0.83–1.1)	1.0 (0.83–1.2)	
Post-operative - Mean (SD)	1.20 (0.79)	1.35 (1.22)	0.143
Median (Q1 – Q3)	1.1 (0.9–1.33)	1.2 (1.01–1.41)	
**Tumor size**			
≤ 3 cm n (%)	487 (62.3)	255 (55.6)	**0.023**
> 3 cm n (%)	295 (37.7)	204 (44.4)	
**Surgical treatment**			
Partial Nephrectomy n (%)	602 (69.7)	295 (59.7)	**< 0.001**
Radical Nephrectomy n (%)	262 (30.3)	199 (40.3)	
**Histologic subtype**			
Clear cell n (%)	401 (64.8)	250 (64.3)	0.921
Non-clear cell n (%)	218 (35.2)	139 (35.7)	
**Nuclear grade** n (%)			
Low grade	224 (23.6)	166 (28.9)	**0.025**
High grade	725 (76.4)	408 (71.1)	
**Extracapsular extension** n (%)			
Yes	81 (10.9)	60 (14.0)	0.140
No	661 (89.1)	368 (86.0)	
**ECOG**			
0–1 n (%)	912 (96.1)	543 (94.6)	0.212
≥ 2 n (%)	37 (3.9)	31 (5.4)	
**Karnofsky**			
≤ 80 n (%)	73 (7.7)	69 (12.0)	**0.006**
> 80 n (%)	876 (92.3)	505 (88.0)	
**ASA classification**			
1–2 n (%)	822 (86.6)	421 (73.3)	**< 0.001**
≥ 3 n (%)	127 (13.4)	153 (26.7)	

* *p*-Value < 0.05 considered statistically significant. Pearson’s chi-squared test or Fisher’s exact test were used in categorical variables when appropriate. Student’s t-test was used in continuous variables

On univariable analysis, extracapsular extension (ECE), ASA classification and bilaterality were associated with recurrence (data not shown). On multivariable model, bilaterality, ECE and ASA classification ≥3 were significant predictors of local recurrence. We did not identify significant predictors for distant metastasis in our series (Table 3).

**Table 3:** Multivariable logistic regression analysis of predictive factors for local recurrence (a) and metastasis during follow-up (b)

**(a) Variable**	**Category**	**OR**	**95% CI**	*p*Value *
**Signs and symptoms at diagnosis**	–	1.265	0.593–2.694	0.543
**Bilaterality**	Bilateral tumors	5.936	2.104–16.746	**0.001**
**Extracapsular extension**		2.700	1.216–5.995	**0.015**
**ASA classification**	ASA ≥ 3	3.380	1.643–6.957	**0.001**
**(b) Variable**	**Category**	**OR**	**95% CI**	*p*Value *
**Gender**	Male	4.494	0.545–37.058	0.163
**Bilaterality**	Bilateral tumors	2.112	0.235–18.954	0.504
**Extracapsular extension**		3.849	0.881–16.821	0.073
**Histologic subtype**	Non-clear cell	Ref.	–	–
	Clear Cell	2.464	0.484–12.531	0.277

* *p*-Value < 0.05 considered statistically significant

## Discussion

Several reports attempt to provide information regarding predictive clinical, pathological and molecular factors in RCC leading to aggressive histology or unfavorable outcomes. Majority of published literature is provided from developed countries. After the propagation of cross-sectional images, incidence of SRM has increased worldwide, as we verified in LA. Some of them may present adverse clinico-pathological features, potentially compromising the oncological outcomes. NSS demonstrates lower rates of chronic kidney disease or cardiovascular events. However, there are some issues about oncological efficacy of PN in comparison to RN [8, 15, 16].

In literature, benign SRM are found in 13 to 30% of the cases and about 40% correspond to low aggressiveness [17–19]. A recent study from Mexico, involving elderly patients, reported 26% of pT1a [20]. Our series evidenced a similar proportion of pT1a cases (25.2%). However, we were not able to report reliable data about benign SRM, because many centers could have rejected information of benign lesions in their database.

The proportion of PN or RN in the surgical treatment of SRM was clearly heterogeneous comparing this decade with the first period of the study (up to 2009). This can be attributable to the development of minimally invasive techniques, the better expertise of the LA referral centers, and the stage migration with the raise of SRM in the last years [21]. Sun et al. [22] described that PN for SRM increased from 4.7% in 1988 to 40.4% in 2008. A recent British study showed more than 90% of NSS performed in T1 tumors [10]. Cases with lower mean age and less comorbidities had PN performed more frequently. Interestingly, the mean operation time in PN was significantly lower than in RN, maybe due to factors related to the patients or to the participating centers (teaching hospitals or communitarian hospitals).

From 1983 to 2002, overall mortality rose in patients with RCC in the USA. In tumors < 2 cm, this rise was 0.07 to 0.2 deaths per 100,000. In tumors between 2 and 4 cm, this rise was from 0.2 to 1.5 deaths per 100,000 US population [23]. Considering only patients with at least 12-months of follow-up, our 5-year and 10-year OS were 94.7 and 81.3%, respectively. Tumor size in our SRM casuistic did not affect the 10-year OS. Interestingly, Jeon [19] pointed that in studies limited to small tumors, they may not find associations with size and favorable histologic features.

Considering that elderly people have more comorbidities and that RN can proportionate a higher risk of renal dysfunction [20, 24], we found that NSS is associated with a better 10-year OS in this population. Russel [25] analyzed patients ≥80 years of age and described a decreased RCC-specific mortality for tumors larger than 3 cm submitted to surgery. A previous series from Mayo Clinic found also an association of RN for pT1a tumors and a decreased OS, but particularly in younger patients (< 66 years of age) [26]. Similarly, in a recent Cochrane review, PN was associated with a reduced time-to-death of any cause (HR: 1.50, 95%CI: 1.03–2.18) [27]. The effect of age on cancer-specific mortality was tested in a recent study. Elderly patients (≥ 70 years) evidenced larger masses, a higher nuclear grade, and presented worse OS and CSS in comparison with those patients < 70 years [28]. Similarly to our study, elderly patients underwent more frequently to RN than PN. Treatment decisions favoring PN in selected SRM in elderly patients should be discussed, particularly if we consider that reference centers have acquired expertise on minimally invasive techniques.

The benefits of regional or retroperitoneal lymphadenectomy are controversial, particularly in small renal masses, that rarely present positive lymph nodes. The extent and number of retroperitoneal lymph nodes removed have no defined templates. Marchioni et al [29] studied pT2-pT3 renal masses treated with RN and evidenced no gain in CSS related to the lymph nodes dissection (LND). In our study, LND was not analyzed because it is not a routine procedure in SRM, even when it is treated with RN.

Ball et al. [30] described that male gender, tumor size ≥3 cm and R.E.N.A.L. nephrometry score ≥ 8 are predictive of unfavorable pathology in cT1a cases. Other recent study with SRM under AS described 2% of distant metastasis during follow-up and associated non-black race, male gender, high nuclear grade, clear cell subtype and tumor size as independent predictors [31]. We were able to find an association of ECE with local recurrence, but not with distant metastasis. ECE is a rare event in SRM, due to this reason, we aggregated perinephric fat invasion, hilar fat invasion, and renal pelvis invasion in this variable. This feature was seldom evidenced and it demonstrated an ominous factor in the follow-up of these patients.

Furthermore, bilaterality and ASA classification ≥3 associated with local recurrence following surgery. Patients classified with ASA ≥ 3 can present more aggressive tumors [32, 33]. Usually, these patients have routine clinical appointments and, probably, perform more image exams than fit people. We suggest that these patients can have earlier diagnosis of recurrence than others.

Regarding nephrometry, patients with totally endophytic tumors performed RN more than three times than patients with exophytic tumors (76% vs. 24%). Due to the historic long-time design of this series, we have no confident data of nephrometry scores (e.g.: R.E.N.A.L.). We did not find differences between totally endophytic or exophytic tumors in OS neither in our recurrence or distant metastasis analysis (data present in only 449 cases).

There are limitations in our study, including the retrospective design and heterogeneity of each center. Pathologic reports were provided by different pathologists and central pathology review was performed only in Brazilian cases (*N* = 244). Furthermore, the heterogeneity of the different centers and the long period of this series could lead to a selection bias of the elderly patients underwent PN or RN. Proposed protocols regarding surgical approaches have been changed during the last decades. Additionally, we did not include patients treated with non-surgical approaches, such as active surveillance, watchful waiting, or ablative treatments, which are possible options in SRM, particularly in elderly or in patients with multiple comorbidities. Due to the lack of data, we were not able to report the nephrometry score of the cases. Related to the secondary outcomes for local recurrence and distant metastasis, we were unable to define the exact moment of the event. Thus, a logistic regression model was the most appropriate for the analysis of secondary outcomes, instead of a survival analysis. Finally, the short median follow-up time of 24 months could be related to possible influences on survival outcomes. Despite these limitations, this study is one of the largest known series of patients with small renal masses in Latin America. Many of the involved institutions are referral centers or teaching institutions. They tend to have skilled uropathologists, more detailed pathology records, and more available data about oncological outcomes. The results are important to recognize particular features of SRM in LA and to counsel patients about appropriate decisions.

## Conclusions

RN is still overly done in the SRM setting in LA. However, NSS procedures are performed in a proportion similar to developed areas and has been increasing in the last years. RN might be considered an overtreatment for many of these patients. Even in elderly individuals, if good functional status, sufficiently fit to surgery, and presenting favorable tumor characteristics, they should be encouraged to underwent PN. Posteriorly, a more intense follow-up routine could be discussed in SRM cases with unfavorable characteristics, intend to an earlier diagnosis of recurrence or distant metastasis.

## Data Availability

The datasets generated and/or analyzed during the current study are not publicly available due to containing information that could compromise research participant privacy.

## Abbreviations

ASA: American Society of Anesthesiologists
BMI: Body Mass Index
ECE: Extracapsular extension
ECOG: Eastern Cooperative Oncology Group
LA: Latin America
LND: Lymph nodes dissection
NSS: Nephron-sparing surgery
OS: Overall Survival
PN: Partial Nephrectomy
PSM: Positive surgical margins
RCC: Renal Cell Carcinoma
RN: Radical Nephrectomy
SRM: Small Renal Mass
